# Advanced adenoma diagnosis with FDG PET in a visibly normal mucosa: a case report

**DOI:** 10.1186/1752-1947-1-99

**Published:** 2007-09-20

**Authors:** Bhavya Rehani, Richard M Chasen, Yvonne Dowdy, Ankur Bharija, Martin Satter, Pamela Strohmeyer, Joseph Mantil

**Affiliations:** 1 Dept. of Nuclear Medicine/PET, Kettering Medical Center, Southern Blvd, Kettering, OH 45429, USA; 2 Dept. of Medicine, Wright State University SOM, Col Glenn Highway, Dayton. OH 45429, USA; 3 Ambulatory Endoscopy Center of Maryland, Van Dusen Road, Laurel, MD 20707, USA; 4 Dept. of Pathology, Kettering Medical Center, Southern Blvd, Kettering, OH 45429, USA; 5 Dept of Internal Medicine, Kettering Medical Center. Southern Blvd, Kettering, OH 45429, USA

## Abstract

**Background:**

An accurate, early diagnosis and treatment of adenomatous polyp can curtail progression to colorectal cancer. F-18 fluorodeoxyglucose positron emission tomography (F-18 FDG PET) reveals the biochemical changes associated with the development of many cancers which precede the appearance of gross anatomical changes that may be visualized during surgical resection or via imaging with MR or CT.

**Intervention:**

We detail the history of a 64 year old female who had a whole-body FDG PET scan as a part of an employee wellness program. A dose of 12.2 mCi of F-18 labeled FDG was administered.

**Results:**

A focal cecal uptake with a standardized uptake value (SUV) of 8.9 was found on the PET scan. Conversely, only normal mucosa was observed during a colonoscopy done 2 months after the PET scan. Motivated by the PET scan finding, the colonoscopist performed a biopsy which revealed a villous adenoma without high grade dysplasia. Pathology from tissue extracted during an exploratory laparatomy completed one month later found the lesion to be a villous adenoma with high grade dysplasia.

**Conclusion:**

Whole-body FDG PET scan revealed the biochemical metabolic changes in malignancy that preceded the appearance of any gross anatomical abnormality. A positive FDG PET scan indicative of colorectal cancer should be followed up with a colonoscopy and biopsy even in a visibly normal mucosa.

## Background

We hereby report a case of advanced adenoma which was detected on a whole-body FDG PET scan despite the fact that the mucosa had a normal appearance on follow up colonoscopy. F-18 fluorodeoxyglucose positron emission tomography (F-18 FDG PET) reveals the biochemical changes associated with the development of many cancers which precede the appearance of gross anatomical changes that may be visualized during surgical resection or via imaging with MR or CT.

## Methods

A dose of 12.2 mCi was given to the patient following a 4 hour fasting period. The tracer incorporated for 1 hour prior to image acquisition. A 6 bed position, whole-body PET scan was performed on a Siemens HR+ PET scanner. Each bed position included a 2 minute transmission followed by a 5 minute emission scan. Reconstruction parameters were 2 iterations/8 subsets (OSEM) with a 4 mm smoothing Guassian filter

## Case presentation

**Figure 1:**
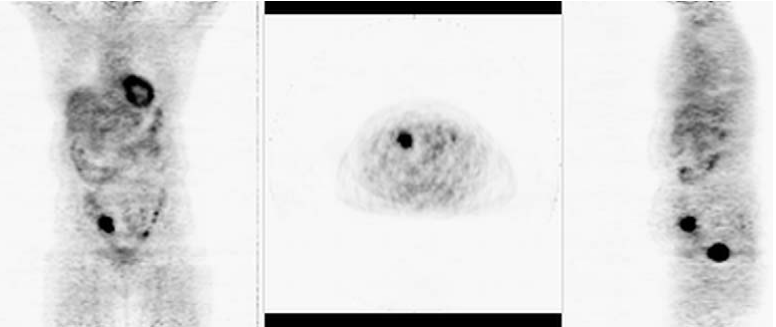
FDG PET scan shows focal abnormality in the cecum with an uptake of SUV of 8.9.

The patient is a 64 year old female who had a whole body FDG-PET scan in June 2004 as part of an employee wellness program. The scan was negative except for a focal abnormality in the cecal area which had an SUV of 8.9 (Figure 1). The patient was completely asymptomatic. A colonoscopy performed two years earlier was normal.

**Figure 2:**
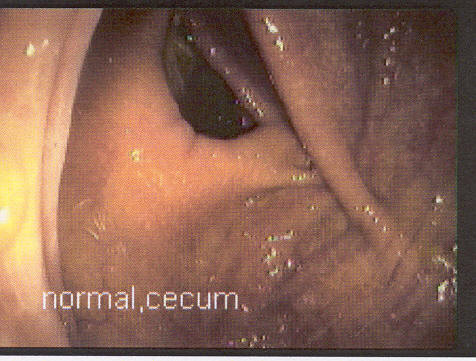
Normal cecum on colonoscopy.

**Figure 3:**
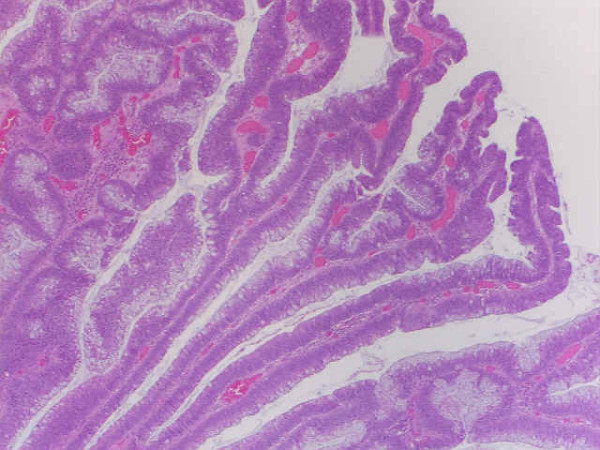
Villous adenoma with high grade dysplasia.

Two months after the FDG-PET scan, a colonoscopy was performed. There was no visible abnormality in the cecum (Figure 2) but prompted by the positive PET scan, a biopsy was obtained. During the biopsy, the gastroenterologist noted abnormal tissue consistency and suspected a blanket of polyps. Multiple biopsies were obtained although no gross abnormality was visible. The pathology results showed a villotubular adenoma without high grade dysplasia. An exploratory laparatomy was performed one month after the colonoscopy to remove the tissue with abnormal histology. Gross pathology showed an exophytic polypoid lesion in the cecal pouch, adjacent to the ileocecal valve. The lesion measured 4.5 × 3 × 0.5 cm. Microscopic examination revealed villous adenoma with focal high grade dysplasia, negative for invasive carcinoma (Figure 3). Surgical margins and lymph nodes were free from dysplasia. A subsequent whole-body FDG PET/CT scan performed 10 months postoperatively was found to be normal.

## Discussion

Polyp is a small growth of tissue shaped like the head or stalk of a mushroom. They are classified histologically as neoplastic (adenomatous) or non neoplastic [1]. Adenomas are traditionally divided into three types: tubular, tubulovillous, and villous. Advanced adenoma is defined by a national polyp study as being >= 1 cm in diameter or containing appreciable villous tissue or high grade dysplasia [2]. Our patient had a villous adenoma with high grade dysplasia. Once diagnosed, advanced adenomas should be resected to prevent progression to cancer and thus early diagnosis is critical.

In this educational case report, the patient had a high grade villous adenoma which was discovered due to its increased FDG uptake on a whole-body PET scan but the mucosa looked normal on the colonoscopy. FDG PET scanning detects biochemical changes which precede gross morphological changes in cancerous tissue. Increased cell surface glucose transporter protein 1 to 5 and increased level of hexokinase pathway explain the high FDG uptake in malignancy.

Narrow Band imaging and Chromoendoscopy with indigo carmine can increase the yield of conventional colonoscopy. [3,4] The reasons cancers may be missed on colonoscopy are attributed to incomplete colonoscopy, poor bowel preparation, misinterpretation of what was seen (mostly flat polyps), failure to carry out adequate biopsy of lesions, and system failures related to follow up investigations in patients who had incomplete colonoscopy [5-8]. However, these conditions are not applicable for this case in which the mucosa was visibly normal on colonoscopy. The gastroenterologist has 25 years of experience in Endoscopy. Two other endoscopists were shown the pictures; they both said they would have assumed a false positive scan and wouldn't have done a biopsy of the area.

According to the current guidelines [9], since a colonoscopy had been performed on this patient two years prior to the PET scan, another colonoscopy would not have been repeated for another eight years. A whole-body FDG PET scan enabled the early diagnosis and removal of the advanced adenoma preventing its progression to invasive cancer in this patient.

FDG PET has a high sensitivity in diagnosing premalignant and advanced colonic neoplasia [10-14]. Van Kouwen et al showed in a large prospective study that the sensitivity increases with size and grade of dysplasia [11]. An interesting experimental study showed induced colonic adenomas and carcinomas may be detected in apparently normal mucosa [15].

## Conclusion

Whole-body FDG PET scan revealed the biochemical metabolic changes in malignancy that preceded the appearance of any gross anatomical abnormality. A positive FDG PET scan with a focal uptake in colon should be considered a significant finding indicative of colorectal cancer and follow up colonoscopy and biopsy should be recommended even in a visibly normal mucosa.

## Competing interests

The author(s) declare that they have no competing interests.

## Authors' contributions

BR drafted the manuscript, BR and PS reviewed the literature,

RC did the colonoscopy; AB admitted the patient and followed up with the patient,

JM and MS were involved with imaging the patient; YD interpreted the pathology report.

All authors read and approved the final manuscript.
